# The Role of Circulating MicroRNAs in Cardiovascular Diseases: A Novel Biomarker for Diagnosis and Potential Therapeutic Targets?

**DOI:** 10.7759/cureus.64100

**Published:** 2024-07-08

**Authors:** Loredana Iacobescu, Andreea-Olivia Ciobanu, Antonio-Daniel Corlatescu, Maya Simionescu, Georgian L Iacobescu, Elena Dragomir, Dragos Vinereanu

**Affiliations:** 1 Cardiology, University of Medicine and Pharmacy "Carol Davila", Bucharest, ROU; 2 Cardiology, University Emergency Hospital, University of Medicine and Pharmacy "Carol Davila", Bucharest, ROU; 3 General Medicine, University of Medicine and Pharmacy "Carol Davila", Bucharest, ROU; 4 Biology, Institute of Cellular Biology and Pathology "Nicolae Simionescu", Bucharest, ROU; 5 Orthopedics and Traumatology, University Emergency Hospital, University of Medicine and Pharmacy "Carol Davila", Bucharest, ROU; 6 Cellular Biology, Institute of Cellular Biology and Pathology "Nicolae Simionescu", Bucharest, ROU

**Keywords:** therapy, diagnosis, biomarkers, cardiovascular diseases, micrornas

## Abstract

MicroRNAs, involved in a large variety of pathological conditions, tend to be potential specific biomarkers in cardiovascular diseases. Moreover, these short, non-coding RNAs, regulate post-transcriptional gene expression and protein synthesis, making them ideal for therapeutic targets. Down-regulation and up-regulation of specific microRNAs are currently studied as a novel approach to the diagnosis and treatment of cardiovascular diseases, such as chronic and acute coronary syndromes, atherosclerosis, heart failure, and arrhythmia. MicroRNAs are interesting and attractive targets for cardiovascular-associated therapeutics because of their stability, tissue-specific expression pattern, and secretion of body fluids. Extended research on their isolation, detection, and function will provide the standardization needed for using microRNAs as biomarkers and potential therapeutic targets. This review will summarize recent data on the implication of microRNAs in cardiovascular diseases, their potential role as biomarkers for diagnosis, and also the challenges of using microRNAs as future therapeutic targets.

## Introduction and background

Cardiovascular diseases remain the main cause of mortality and morbidity in industrialized countries, with high costs for the healthcare systems [1]. Significant therapeutic improvements have been made in the last decades by surgical, interventional, and pharmacological approaches [2]. Meanwhile, continuous scientific efforts have been made to discover the underlying subtle mechanisms of a variety of cardiovascular diseases, such as chronic and acute coronary syndromes, atherosclerosis, heart failure, and arrhythmias, to develop new biomarkers for diagnosis, as well as new therapeutic targets [3].

In 1993, the research community made the first discoveries of the function and importance of small RNAs and their role in controlling gene expression in humans, labeling them microRNAs (miRs) [4]. Since then, microRNAs have been intensively studied for their promising role as diagnostic and prognostic biomarkers in various diseases and their involvement in development, cell differentiation, proliferation, and apoptosis. These research studies connected microRNAs to mechanisms and diagnosis of cancer, neurodegenerative, and cardiovascular diseases, and also to therapy as a potential gene-specific therapeutic target [4-6].

MicroRNAs are single-stranded, non-coding RNA molecules of approximately 22 nucleotides that negatively regulate gene expression at the post-transcriptional level by inhibiting mRNA translation and promoting mRNA degradation [7]. The functions of microRNAs are heightened in various diseases, which makes them possible candidates for therapeutic manipulation [3,7]. In cardiovascular diseases, the first report on microRNA implication was published in 2006, in which van Rooij et al. demonstrated that signature patterns of microRNA expression correlated with heart failure and cardiac hypertrophy in mice and humans [8]. Since then, more and more studies have investigated changes in microRNA expression in diseased human hearts and vascular tissues, their gain and loss of function, and their involvement in cellular processes, such as cell survival (miR15), extracellular matrix production (miR21 and miR29), and hypoxia (miR210) [3,9,10].

This review will summarize recent data on the implication of microRNAs in cardiovascular diseases, their potential role as biomarkers for diagnosis, and the challenges of using microRNAs as future therapeutic targets.

## Review

MicroRNAs in ischemic heart disease and atherosclerosis

Atherosclerosis is a chronic inflammation linked to endothelial injury and an imbalance of lipid metabolism [11]. The involvement of microRNAs in atherosclerosis has been widely studied, including processes such as signaling, migration and proliferation of vascular smooth muscle cells, apoptosis, motility, and plaque angiogenesis [4,7]. Endothelial cells, smooth muscle cells, macrophages, and all other cellular components that are involved in plaque and thrombus formation can release more or less microRNAs in the circulation, and this imbalance can serve as biomarkers for early atherosclerosis [12]. Table 1 shows the role of microRNA in atherosclerotic plaque development and ischemia or reperfusion injury.

**Table 1:** MicroRNAs in atherosclerotic and ischemia processes. LDL: low-density lipoprotein; HDL: high-density lipoprotein; VSMC: vascular smooth muscle cells; VCAM1: vascular cell adhesion molecule 1; SPRED1: Sprouty-related EVH1 domain-containing protein 1; DLK1: delta-like homolog 1.

MicroRNA	Process implication	Specific role
miR1/106, miR34a	Ischemia/reperfusion injury	Stress-induced apoptosis; increases arrhythmogenesis [3]
miR21	Atherosclerosis (endothelial cell activation)	Decreases apoptosis of endothelial cells [7]; promotes fibrosis [3]
miR29	Post-infarction remodeling	Down-regulation determines the increased expression of collagens [3,5]
miR155, miR124, miR146	Atherosclerosis (monocyte activation and macrophage maturation)	Mediate inflammatory macrophage response and monocyte activation [2,7]
miR122, miR33, miR133	Cholesterol biosynthesis	miR122 raises LDL cholesterol levels, whereas miR33 decreases HDL cholesterol levels [7,11]
miR210 miR126 miR92a	Proangiogenic	Influence process of leucocyte infiltration and adhesion to endothelium [3]; modulate plaque angiogenesis [7]
miR208, miR499	Cardiac remodeling	Inhibits mitochondrial apoptosis and protects from oxygen free radicals injury in cardiomyocytes [7,8]
miR221, miR143, miR195	Fibrous cap stabilization and VSMC proliferation	Induce VSMC proliferation; stabilize the plaque [7,9]
miR145, miR17	Inflammation down-regulation	Produce VSMC differentiation and VSMC phenotypic switching [9]
miR126, miR92a	Reduce angiogenesis, inflammation, and apoptosis for cardiovascular protection	Inhibit angiogenesis and endothelial cell proliferation, targeting VCAM1, SPRED1, and DLK1 [8,9]

Several studies have shown that microRNAs, such as miR1/106, miR133, miR21, miR34a, miR146a, miR146b, and miR210, are implicated in ischemic heart disease and are up-regulated in the atherosclerotic plaques [12,13]. miR155 is specifically expressed in the atherosclerotic plaque and the pro-inflammatory macrophages. In a study that screened 667 microRNAs in patients with acute myocardial infarction (AMI), miR155 was among 11 microRNAs highly expressed in patients at high risk of cardiac death [14]. Xie et al., in 159 patients who underwent percutaneous coronary intervention after AMI, have shown that decreased levels of miR155, associated with increased target gene SH2-containing inositol 5’-phosphatase 1 (SHIP-1) expression, determine a low incidence of periprocedural myocardial infarction, with lower levels of cardiac troponin I. Meanwhile, they determine less inflammatory cytokines (interferon-gamma, tumor necrosis factor-alpha, IL6) expression after rosuvastatin treatment [14,15].

miR208a, miR499, miR1, and miR133 have high levels in the plasma of patients with AMI, where apoptotic cell death plays an important role in the detrimental myocardial changes [12,16]. Interestingly, miR1 and miR133 are involved in the regulation of apoptosis in cardiomyocytes in opposing actions, miR1 being pro-apoptotic, whereas miR133 is anti-apoptotic, suggesting the potential role of miR1 in increased apoptotic cell death and arrhythmogenicity in myocardial infarction [17]. miR21 is the only microRNA that is gradually up-regulated in both cancer and cardiovascular diseases, and it was found to prevent cell damage when exposed to oxygen free radicals [18,19].

**Figure 1:**
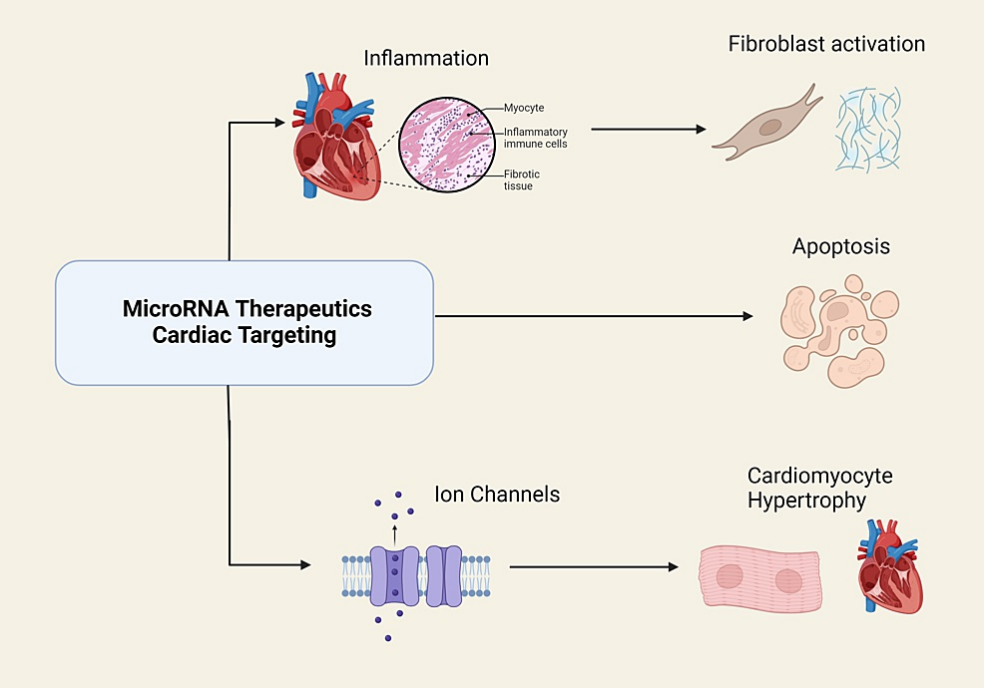
MicroRNA therapeutics targeting cardiac stress pathways. This diagram illustrates the impact of microRNA therapeutics on various aspects of cardiac stress and the associated cellular processes. The figure was created by the authors.

By comparison to healthy controls, in patients with coronary artery disease, a down-regulation of inflammation-related miR145 and miR17 was found, which consequently reduced the circulating levels of angiogenesis-related miR126 and miR92a [19]. It was also shown in mice models with myocardial infarction that systemic administration of an antagomir, which inhibits miR92a, produces blood vessel growth and functional recovery of the damaged cardiac cells, proving its effect on angiogenesis [20]. Wang et al. showed that miR1, miR133a, and miR208b were significantly increased in AMI compared to unstable angina, in a study that included 33 patients with non-ST elevation AMI and 33 patients with chronic coronary syndrome [21]. Moreover, they found that miR208b has a higher specificity (100%) and sensitivity (91%) for the diagnosis of AMI (area under the curve (AUC): 0.965; 95% CI: 0.920-1.000), compared to miR1 and miR133a, that had a sensitivity of only 33% and 15%, respectively [18]. It has been suggested that miR208a might be a novel biomarker detectable in the plasma of patients with AMI within one to four hours from symptom onset when TnT is still below the cutoff value [21]. miR146 is another microRNA proved to be involved in the inflammatory process of atherosclerosis by reducing the production of pro-atherogenic cytokines and, therefore, decreasing plaque development (Figure 1) [22].

MicroRNAs in heart failure

In heart failure, the remodeling process and hypertrophic growth are adaptive mechanisms. Consequently, intracellular signaling pathways are activated, and transcriptional mediators are released in cardiac myocytes. Recent studies suggested the possible role of microRNA in heart failure [13]. Thus, Tijsen et al. found seven microRNAs in the plasma of patients with heart failure: miR423-5p, miR18b, miR129-5p, miR-1254, miR675, miR622, and miR202 [23,24]. Mature miR423-5p correlated with the clinical diagnosis of heart failure, with a receiver operating characteristic (ROC) curve showing an AUC of 0.91 (95% CI: 0.84-0.98) [24]. Moreover, the circulating levels of miR423-5p and miR18b were associated with disease severity with a negative correlation with left ventricle ejection fraction (LVEF) and higher levels in patients with a higher New York Heart Association (NYHA) functional class [24,25].

It can be concluded that the high level of some microRNAs in patients with heart failure is related to the severity of the disease, although it is not very clear which cell type is responsible for the release of those miRNAs in plasma and also how are they released into circulation; these are aspects that need more studies to be clarified [3,25]. Goren et al. showed in their study that increased levels of miR320a, miR-22, and miR-92b correlated with elevated brain natriuretic peptide (BNP) levels, a wide QRS complex on ECG, and left ventricular and left atrial dilation on echocardiography. These microRNAs were also significantly higher in patients with heart failure with reduced ejection fraction compared to the control healthy group [26,27]. Meanwhile, Fukushima et al. showed that miR126, also known as “angiomiR,” expressed only in endothelial cells, correlated negatively with age (r2 = 0.52; P = 0.0006; N = 17) and BNP levels (r2 = 0.25; P = 0.0003) in the NYHA II-IV groups. They also evaluated the circulating values of miR126 in patients with heart failure NYHA IV class and then when they improved to NYHA III, they found that plasma levels of miR126 were upregulated [28], which might indicate its possible role as a biomarker of heart failure [28,29]. Another microRNA involved in the progression of heart failure is miR155, which was found to be high in patients with heart failure compared to healthy controls, and it also correlated positively with the left ventricular mass index [14,30]. Moreover, He et al. demonstrated the role of miR155 in cardiac remodeling after experimental AMI, in which loss of miR155 in fibroblasts protected against left ventricular dysfunction, by targeting tumor protein p53-inducible nuclear protein 1 (TP53INP1) [31]. In their study, they knocked down TP53INP1 using microRNA technology and evaluated cell proliferation and gene alterations in cardiac fibroblasts treated with transforming growth factor-beta (TGFβ). The results were a decreased expression of TP53INP1 by microRNA in mice cardiac fibroblast. Moreover, the levels of collagen I/III were significantly increased in the TP53INP1 group compared to the control group, while the levels of caspase-3 were significantly lower, meaning an increased cell proliferation and proving that miR155 regulates cardiac fibroblast differentiation by targeting TP53INP1 [31,32]. In human hypertrophic heart, it was shown that miR195, a cardiac-specific microRNA, is up-regulated; meanwhile, in mice, its overexpression leads to dilated cardiomyopathy and heart failure [33].

MicroRNAs in arrhythmias

Cardiac arrhythmias remain a major health problem due to their unpredictable evolution and their potential for sudden cardiac death [33]. The main mechanisms involved in the electrical disorder, leading to cardiac arrhythmias, are impaired excitation and/or conduction, enhanced automaticity, and abnormal repolarization. Thus, cardiac arrhythmias appear either in proarrhythmic conditions, such as cardiac remodeling or myocardial ischemia, or malfunction of the ion channels, due to gene mutations of the channel proteins [34]. Studies have shown that microRNAs are involved in mediating arrhythmogenesis, and their role in regulating the expression of cardiac ion channels suggests their potential to develop novel antiarrhythmic agents [35]. Yang et al. studied miR1 in rat models with AMI, as well as in patients with coronary artery disease. They found significantly increased expression of miR1 in the myocardium of patients with coronary artery disease [13,35]. Moreover, in ischemic rats, administration of miR1 increases arrhythmogenicity, whereas reducing miR1 by using its specific inhibitor, antisense oligonucleotides, suppresses arrhythmias. This proves that miR1 has pro-arrhythmic as well as arrhythmogenic effects [33,35]. Pro-arrhythmogenic effect of miR1 is based on the upregulating of miR1 expression during myocardial infarction and, consequently, post-transcriptional repression of the genes for the ion channels GJA1 and KCNJ2, which finally leads to conduction slowing and ischemic arrhythmias [35,36].

MiR133 is also involved in the arrhythmogenic processes [36]. It has been shown that miR133 is upregulated in the diabetic rabbit heart, while exogenous administration of miR133 into diabetic rabbit myocytes determines post-transcriptional repression of ERG (ether-a-go-go-related gene), a long QT syndrome gene that encodes a K+ channel [31,36]. Suppression of ERG produces important depression of Ikr, which determines slowing the repolarization and prolonged QT [36,37]. Meanwhile, downregulation of miR1 and miR133 is associated with an increased level of protein HCN2 and HCN4, two important cardiac pacemaker channel proteins, involved in automaticity and arrhythmia [38]. Xiao et al. demonstrated that gene-specific miRNA mimics, designed to target the 3′ untranslated regions (3′ UTRs) of HCN2 and HCN4, determined the abrogation of the function of these two channels, while miRNA-masking antisense significantly increased HCN2/HCN4 expression and function, providing possible novel gene therapy strategies for cardiac arrhythmias [39].

Atrial fibrillation (AF) is one of the most commonly encountered arrhythmias, associated with a high risk of mortality and morbidity [13]. Many experimental studies determined the regulatory function of microRNAs at the post-transcriptional level. However, there are few clinical trials regarding the implication of circulating microRNAs in AF by comparison with their implication in coronary artery disease or heart failure. There are though small cohort studies that suggested the involvement of some microRNAs in the pathogenesis of AF: miR34a, miR133, miR590, miR328, and miR21 [11,13,34]. Thus, Zhu et al. showed that miR34a inhibits the expression of ankyrin B (Ank-B), an adaptor protein associated with AF. In their study, miR34a was upregulated in patients with AF, decreasing the level of Ank-B, by comparison to subjects in sinus rhythm, which might have an important role in the early electrophysiological changes and development of AF through the regulation of the Ank-B expression [40]. Meanwhile, it has been shown that miR133 and miR590 promote atrial remodeling, which leads to AF [13]. Moreover, the downregulation of miR328 was associated with atrial remodeling, probably through targeting the L-type Ca channels [41], while higher levels of miR21 are associated with atrial fibrosis in AF [42].

MicroRNAs and cardiac fibrosis

Myocardial fibrosis is a major complication of cardiac diseases, related to the abnormal deposition of collagen by cardiac fibroblasts. The pathophysiology includes cardiomyocyte apoptosis and the release of profibrotic factors, processes regulated by microRNAs [43]. Excessive accumulation of extracellular matrix protein determines modification of electrical properties and mechanical stiffness, leading to arrhythmias and diastolic dysfunction and, finally, to the progression of heart failure [13]. There are several microRNAs, such as miR133, miR30c, miR29, and miR21, involved in the remodeling of the extracellular matrix, through regulating connective tissue growth factor (CTGF), a key molecule of the fibrosis process [13,44]. Thus, high levels of miR133 and miR30c in cultured fibroblasts lead to a suppression of CTGF and, consequently, lower production of collagen [44-46]. Moreover, miR133 and miR30c are down-regulated in patients with heart failure and cardiac hypertrophy, promoting fibrosis [46] MiR29, highly expressed in cardiac fibroblasts, has been found to be down-regulated in the peri-infarcted zone in rats [43]. Meanwhile, injection of cholesterol-modified oligonucleotides (antagomir miR29b) determined a high collagen expression in the heart, whereas over-expression of miR29 in fibroblasts reduced the collagen level. This suggests that miR29 might be associated with cardiac fibrosis, as a negative regulator of collagen expression in the infarcted heart [47]. Thum et al. showed that miR21 promoted cardiac fibrosis in an experimental heart failure model [48]. They found that miR21 levels were high in fibroblasts of the failing heart, increasing the mitogen-activated protein kinase (MAPK) and, therefore, increasing the secretion of growth factors and survival of fibroblasts. These lead to extended fibrosis and cardiac hypertrophy. On the contrary, injection of a specific miR21 antagomir in a mouse model induced a reduction of gene encoding collagens and extracellular matrix, with a reduction of MAPK activity, inhibition of interstitial fibrosis, and improvement of cardiac function [13,48,49].

Recent studies showed that miR101 and miR101a have an important role in the suppression of cardiac fibrosis in a rat model of myocardial infarction. This is due to their effect on the downregulation of TGFβ, through targeting TGFβ receptor type 1 (TGFβr1) [50]. MiR122 seems to be also associated with cardiac fibrosis and, therefore, with cardiac dysfunction, decreased left ventricular ejection fraction, and increased levels of N-terminal pro-B-type natriuretic peptide (NT-proBNP) [51,52].

Therapeutic strategies based on microRNAs

Currently, therapeutic perspectives of microRNAs have two approaches: overexpression or upregulation of a specific microRNA, and inhibition or down-regulation of a specific microRNA [20]. Thus, the use of microRNA mimics determines a high level of microRNA, with suppression of gene expression, whereas the use of anti-microRNAs determines a low level of microRNA, with activation of gene expression. The chemistry behind gene alterations through antisense technology is significantly more advanced than microRNA overexpression through mimicry. The main therapeutic roles of microRNAs in cardiovascular diseases are presented in Table 2.

**Table 2:** Role of microRNAs as therapeutic targets in cardiovascular diseases. miRNA: microRNA; LVEF: left ventricle ejection fraction; HDL: high-density lipoprotein.

Cardiovascular disease	microRNA	Therapeutic role	AntimiR/mimics
Acute myocardial infarction	miR210 and miR34a	Reduced apoptosis and promoted neovascularization	AntimiR
Acute myocardial infarction	miR29b	Reduced fibrosis after myocardial infarction	AntimiR
Acute myocardial infarction	miR15	Inhibited cardiac remodeling	AntimiR
Acute myocardial infarction	miR590	Improved LVEF and reduced infarction size	AntimiR
Acute myocardial infarction	miR199a	Improved LVEF and reduced infarction size	Mimics
Heart failure	AntimiR208a	Improved cardiac function	AntimiR
Heart failure	miR208a	Increased cardiac hypertrophy	Mimic
Heart failure	miR133	Reduced cardiac hypertrophy and fibrosis	AntimiR
Heart failure	miR199b	Improved LVEF	AntimiR
Heart failure	miR132-3p		AntimiR
Atrial fibrillation	miR328	Reduced left atrium remodeling	Mimic
Atherosclerosis	miR21	Reduced vascular smooth muscle cell proliferation, stimulated apoptosis, increased level of HDL; reduction of cholesterol level	AntimiR
Atherosclerosis	miR33	Reduced vascular smooth muscle cell proliferation, stimulated apoptosis, increased level of HDL; reduction of cholesterol level	AntimiR
Atherosclerosis	miR122	Reduced vascular smooth muscle cell proliferation, stimulated apoptosis, increased level of HDL; reduction of cholesterol level	AntimiR

Anti-microRNAs (anti-miRs) are chemically modified antisense oligonucleotides that carry the complementary reverse sequence of a mature miRNA to improve binding affinity, nuclear resistance, and cellular uptake, and reduce levels of a pathogenic miRNA [37,53,54]. AntagomiRs are a class of anti-miRs, conjugated to cholesterol, to increase tissue intake of antisense inhibitor oligonucleotides and to facilitate cellular uptake. They can strongly inhibit the expression of target miRNAs after in vivo injection, as shown by Krutzfeldt et al. [37] They used an antagomiR for miR122, a liver-specific microRNA involved in lipid metabolism and hepatitis C virus replication. The result was a significant reduction of cholesterol levels in mice [37,55]. Rayner et al. studied antimiR33, which is also involved in lipid metabolism. They showed that after being injected in mice, it increased levels of HDL cholesterol and, consequently, decreased the progression of atherosclerosis. This might become an important therapeutic strategy [56].

Carè et al. performed one of the first studies using an antagomir to inhibit miR133, involved in cardiac hypertrophy. They injected subcutaneous osmotic minipumps in mice, which delivered a cholesterol-based anti-miR133, and they observed that after one month there was a higher degree of cardiac hypertrophy, as assessed by echocardiography. Accordingly, miR133 mimics might be a relevant therapeutic agent for the regression of left ventricular hypertrophy [19,56].

In heart failure, antagomiRs have also shown their benefit by reducing cardiac hypertrophy and fibrosis in mice models. Thus, inhibition of miR199b through intraperitoneal administration of antagomir, in mice, normalizes the activity of calcineurin/nuclear factor of activated T cells and, consequently, reduces cardiac hypertrophy and fibrosis. Meanwhile, inhibition of miR208a by subcutaneous administration of antimiR208a, in hypertensive rats, produced an inhibition of left ventricular remodeling and pathological myosin switching, with an overall improvement of cardiac function and survival [55].

Recently, in a first-in-human phase 1b randomized, double-blind, placebo-controlled study, Täubel et al. showed that CDR132L, a specific antisense oligonucleotide, combined with a first-in-class miR132-3p inhibitor, was safe and well tolerated. They produced the reduction of NT-proBNP levels and improvement of LVEF, which suggests the prevention of cardiac remodeling in patients with heart failure. Moreover, inhibition of miR15, upregulated in an ischemic heart, determines the reduction of the infarct size, inhibition of cardiac remodeling, and a significant improvement of LVEF at two weeks of echocardiography evaluation in mice [9].

While microRNA inhibition involves stable single-stranded oligonucleotides, microRNA mimic technology is more challenging, due to the difficulty of loading the mimic into the RNA-induced silencing complex (RISC) [57]. MicroRNA mimics are double-stranded oligonucleotides with a guide strand, identical to the mature microRNA, and a complementary or partially complementary passenger strand. The guide strand acts as the endogenous microRNA, and blocks targeted gene expression, while the passenger strand is linked to cholesterol to produce cellular uptake [58]. These RNA fragments enhance the expression of microRNAs that are downregulated in cardiovascular diseases. Viral approaches, especially adeno-associated virus-mediated microRNA gain of function, are widely used, compared to few in vivo applications of microRNA mimics.

It has been proved that miR21 levels are downregulated in the infarcted areas. Dong et al. showed that transfection with adenovirus expressing miR21 in rats, after induced myocardial infarction, determined cell apoptosis, reduced infarction size, and improved left ventricular ejection fraction [58]. In another study, Pan et al. showed that using a miR101 mimic determined a higher left ventricular performance, expressed by higher ejection fraction and fractional shortening, and an antifibrotic effect, by influencing the endothelial extracellular matrix deposition, in an experimental model on mice, in which left descending coronary artery was performed to induce cardiac infarction. Their results show that the downregulation of miR101a after cardiac infarction inhibited the proliferation of cardiac fibroblasts and the production of collagen [59].

Another strategy of upregulation of microRNAs is the transplantation of mesenchymal stem cells, in an experimental mouse model with myocardial infarction, leading to improved cardiac function by enhanced myocyte differentiation [60].

There are few recent studies that proved the role of microRNA mimics in inducing cardiac regeneration, being well known that cardiomyocytes have a limited proliferative and regenerative capacity after myocardial infarction. Thus, the administration of miR590 and miR199a mimics produced differentiated cardiomyocytes by their re-entry into the cell cycle. Furthermore, using an adeno-associated virus vector, the overexpression of miR590 and miR199a produced an improvement in left ventricular ejection fraction and a reduction of infarction size [60].

## Conclusions

Since their discovery in the 1990s, microRNAs have been shown to play important roles in various cardiovascular diseases, such as atherosclerosis, arrhythmias, acute coronary syndromes, and heart failure. A noncoding portion of the genome is critical in the regulation of multiple biological processes, such as differentiation, post-transcriptional regulation of gene expression, and epigenetic regulation. There are numerous studies that reported microRNAs as promising new diagnostic and prognostic biomarkers. However, the validity of applying microRNAs for this purpose is limited by heterogeneous results and low reproducibility. Therefore, larger, unbiased studies are needed to establish their role as biomarkers. The use of antagomirs and miR mimics has already found its way into clinical trials, and promising results show the potential future application of microRNA therapeutics in cardiovascular diseases.
